# Predicting Antifouling Activity and Acetylcholinesterase Inhibition of Marine-Derived Compounds Using a Computer-Aided Drug Design Approach

**DOI:** 10.3390/md20020129

**Published:** 2022-02-08

**Authors:** Susana P. Gaudêncio, Florbela Pereira

**Affiliations:** 1 Associate Laboratory i4HB—Institute for Health and Bioeconomy, NOVA School of Science and Technology, NOVA University of Lisbon, 2819-516 Caparica, Portugal; s.gaudencio@fct.unl.pt; 2 UCIBIO—Applied Molecular Biosciences Unit, Department of Chemistry, Blue Biotechnology and Biomedicine Lab, NOVA School of Science and Technology, NOVA University of Lisbon, 2819-516 Caparica, Portugal; 3 LAQV, Department of Chemistry, NOVA School of Science and Technology, NOVA University of Lisbon, 2829-516 Caparica, Portugal

**Keywords:** marine natural products (MNPs), blue biotechnology, quantitative structure–activity relationship (QSAR), machine learning (ML) techniques, computer-aided drug design (CADD), molecular docking, virtual screening, antifouling activity, acetylcholinesterase enzyme (AChE)

## Abstract

Biofouling is the undesirable growth of micro- and macro-organisms on artificial water-immersed surfaces, which results in high costs for the prevention and maintenance of this process (billion €/year) for aquaculture, shipping and other industries that rely on coastal and off-shore infrastructure. To date, there are still no sustainable, economical and environmentally safe solutions to overcome this challenging phenomenon. A computer-aided drug design (CADD) approach comprising ligand- and structure-based methods was explored for predicting the antifouling activities of marine natural products (MNPs). In the CADD ligand-based method, 141 organic molecules extracted from the ChEMBL database and literature with antifouling screening data were used to build the quantitative structure–activity relationship (QSAR) classification model. An overall predictive accuracy score of up to 71% was achieved with the best QSAR model for external and internal validation using test and training sets. A virtual screening campaign of 14,492 MNPs from Encinar’s website and 14 MNPs that are currently in the clinical pipeline was also carried out using the best QSAR model developed. In the CADD structure-based approach, the 125 MNPs that were selected by the QSAR approach were used in molecular docking experiments against the acetylcholinesterase enzyme. Overall, 16 MNPs were proposed as the most promising marine drug-like leads as antifouling agents, e.g., macrocyclic lactam, macrocyclic alkaloids, indole and pyridine derivatives.

## 1. Introduction

Marine biofouling is the undesired accumulation of micro-organisms, e.g., bacteria, cyanobacteria, unicellular algae and protozoa, and macro-organisms, e.g., seaweeds, barnacles, mussels and shells, on artificial water-immersed surfaces in a dynamic process that starts immediately after water submersion and can be a fast or slow process taking only hours or months to develop, respectively [1]. Marine biofouling creates risks to various industries, such as aquaculture and shipping, as well as for non-marine industries, e.g., paper manufacturing, food processing, underwater construction, power plants and others [2,3]. Settlement on the vessel’s hull results in damage to the rudder and propulsion systems [2,4], leads to an increasing drag of up to 60%, as well as a fuel consumption increase by 40%, increasing carbon dioxide and sulfur dioxide emissions [5] and the spread of nonindigenous marine species into ecosystems worldwide, leading to environmental imbalances [6,7,8,9,10]. The most effective antifouling (AF) coatings contained biocides, such as tributyltin (TBT) and tributyltin oxide (TBTO), which were found to be harmful to non-target organisms and the environment [11] and thus were prohibited by the International Maritime Organization from Ship Surfaces in 2008, generating the demand for new generations of non-toxic or environment-friendly AF solutions [12,13,14].

Natural alternatives including primary or secondary metabolites isolated from marine organisms have been reported in several reviews to inhibit the settlement of different biofouling species [15,16,17,18,19,20,21,22,23,24]. The search for AF agents from marine sources began with bromo-derived metabolites, among the 2-furanone bromine derivatives extracted from red algae, which have been reported to prevent fouling [25], as well as bromopyrrole alkaloid derivatives with AF activity isolated from sponges (oroidin), inspiring the design of more than 50 synthetic analogues [26,27], and, more recently, antifouling bromotyrosine derivatives of the synoxazolidinone and the pulmonarin families [28]. Several studies reported MNPs with antifouling activity comprising the 2,5-diketopiperazine scaffold isolated from the marine sponge *Geodia barretti* [29], 6-benzyl and 6-isobutyl 2,5-diketopiperazine derivatives from marine-derived actinomycete *Streptomyces praecox* [30] and five diketopiperazines, cyclo-(L-Leu-L-Pro), cyclo-(L-Phe-L-Pro), cyclo-(L-Val-L-Pro), cyclo-(L-Trp-L-Pro) and cyclo-(L-Leu-L-Val), from deep-sea *Streptomyces fungicidicus* [31]. Comprising a meroterpenoid scaffold, napyradiomycin derivatives, isolated from marine-derived actinomycetes *Streptomyces aculeolatus*, were investigated by our group as antifouling inhibitors, having the advantage of inhibiting both micro- (antibiofilm activity) and macrofouling [32,33,34].

Computer-aided drug design (CADD) approaches have been used to guide decisions concerning the in vivo and in vitro testing of isolated NPs and extracts [35,36,37,38,39], to assist in the design of bioactive NP derivatives [40,41] and to virtually screen databases of known or proposed NPs [40,42,43,44]. To the best of our knowledge, the antifouling activity was quantitative structure–activity relationship (QSAR) modeled in only two previous works for the settlement of *Mytilus galloprovincialis* larvae [45,46]. Almeida et al. built two QSAR models using multilinear regression methods with, respectively, 19 and 16 nature-inspired (thio)xanthone [46] and chalcone [45] derivatives, including in vitro antifouling activity screening assays for the settlement of *Mytilus galloprovincialis* larvae. 

Acetylcholinesterase (AChE) inhibitors are a class of drugs used for the treatment of Alzheimer’s disease, glaucoma and autoimmune disorders [47,48,49]. The enzymes AChE [28] and tyrosinase (Tyr) were associated with the adhesive processes in the settlement of different biofouling species [28,46,50]. Almeida et al. reported a molecular docking study conducted by modulation of *Electrophorus electric* (fish) AChE of the two most promising (thio)xanthone antifouling agents [46]. Recently, Arabshahi et al. [50] reported an extensive virtual *Tetronarce californica* (fish) AChE homology screening campaign for 10,000 small organic molecules from the Chembridge library. The authors also reported the experimental screening of the most promising AChE inhibitors proposed by the in silico model, against five microfouling marine bacteria and marine microalgae macrofouling tunicate *Ciona savignyi*, discovering a potent novel inhibitor of tunicate settlement [50].

Herein, we report comprehensive computational modeling for the prediction of antifouling activities from two MNP libraries, by employing structure- and ligand-based CADD methodologies. The two libraries comprised 14,492 MNP from Prof. Encinar (http://docking.umh.es/downloaddb, accessed on 25 October 2021) and 14 MNPs from the clinical pipeline of MNPs (eight drugs approved and six MNPs in Phase II and III clinical trials). All the MNPs from the virtual screening libraries that were predicted to belong to the active class, i.e., 125 MNPs, were selected to proceed to the CADD structure-based method, where 125 MNPs selected by QSAR approach were screened by molecular docking against the AChE enzyme. In this CADD approach, a virtual screening hit list comprising 19 MNPs was assented based on some established thresholds, such as the probability of being active in the best antifouling model and the prediction of affinity between the AChE of selected MNPs by molecular docking. A total of 16 MNPs have been proposed as the most promising marine drug-like leads as antifouling agents.

## 2. Results and Discussion

### 2.1. Chemical Space of the Antifouling Model

The whole data set (i.e., 141 small organic molecules) was randomly divided into a training set of 127 molecules (comprising 57 active and 70 inactive molecules) and a test set of 14 molecules (comprising six active and eight inactive molecules), which were used for the development and external validation of the QSAR classification models, respectively. The whole data set comprised seven structural classes or scaffold types, which are represented in Table 1 along with their antifouling activity classes and scaffold representative. 

All seven structural clusters (I, acyclic derivative, II, *O*-heterocyclic derivative, III, *N*-heterocyclic derivative, IV, terpenoid derivative, V, diketopiperazine derivative, VI, chalcone derivative, and VII, miscellaneous) were well represented in the training set, each comprising more than 10 molecules per class. The active class was more represented in three structural clusters with a percentage higher than 50%, namely I—acyclic derivative (100%), III—*N*-heterocyclic derivative (74%) and V—diketopiperazine derivative (67%). In the test set, only five structural clusters were represented, II-V and VII. In Table 1, the most representative scaffolds of the structural cluster are highlighted—for instance, for cluster I, a polyacetylene derivative; II, a chromone and a xanthone derivative; III, a pyrrole and a piperidine derivative; IV, a sesquiterpene derivative; V, a diketopiperazine, VI, a chalcone derivative; and VII, various scaffolds such as peptides and nature-inspired sulfated compounds. All clusters for the training and test sets, except for cluster VII, had an average MW value of less than 500 Da. 

### 2.2. Establishment of QSAR Classification Model

Random Forests (RF) [51] were used to build models for antifouling prediction, exploring well-established PaDEL fingerprints (FPP and descriptors, e.g., five different types of FPs with different sizes (166 MACCS, MACCS keys; 307 Substructure; 881 PubChem fingerprints; 1024 CDK, circular fingerprints; 1024 CDK Ext, extended circular fingerprints with additional bits describing ring features) and 1376 1D&2D molecular descriptors (including electronic, topological and constitutional descriptors)) [52]. The performance of the models was successfully evaluated by internal validation (out-of-bag, OOB, estimation on the training set); see Table 2.

From the seven sets of FPs and descriptors used to build the QSAR classification model, the best set for each type, fragment FPs (Sub), circular FPs (ExtCDK) and molecular descriptors (1D&2D), were selected for further investigations; see Table 2. The 3D descriptors had a well-established relationship with biological activity and were expected to increase both the accuracy and robustness of the predictive models. After the exploration of models derived with molecular descriptors and FPs, we investigated the inclusion of 3D descriptors such as radial distribution function (RDF) descriptors (using a range of 128 and partial atomic charge as an atomic property) and the selection of descriptors using the RF descriptor importance parameter for the best three sets (Sub FPs, ExtCDK FPs and 1D&2D descriptors). Three sets of descriptors (Sub + RDF, ExtCDK + RDF and 1D&2D + RDF) as well as their selection were explored for modeling the antifouling activity using the RF algorithm in Table 3, where the results for the training set in OOB estimation are presented.

The 200 most important descriptors selected by the MeanDecreaseAccuracy parameter of the 1D&2D + RDF model were identified by the RF algorithm and enabled the training of a new RF model with better prediction accuracy in accordance with the Q and MCC values than the model trained with the whole set of descriptors (Table 3). A comparison of three machine learning (ML) techniques using the Weka software (support vector machines, SVM), R software (RF) and Keras software (deep learning multilayer perceptron networks, _d_MLP) for building the antifouling models with the 200 descriptors that were selected by the RF is shown in Table 4 for the test set.

The best models were accomplished with the RF and _d_MLP algorithms using the 200 1D&2D + RDF selected descriptors, which achieved, for both models, a Q and MCC of 0.714 and 0.417 for the external test set. Majority voting predictions (consensus) were obtained by the RF, SVM and _d_MLP models (the consensus model, CM), and did not improve the results, with a Q and MCC of 0.571 and 0.167 for the test set; thus, in the next step of the virtual screening, we used the best model obtained, RF, with the 200 selected descriptors; see Table 3 and Table 4).

The results obtained by the RF for the training and test sets that were in accordance with the seven structural clusters (I–VII), reported in Table 1, are shown in Table 5.

There were three structural clusters (I, II and IV, bold highlighted in Table 5) in which the predictions obtained were better than those obtained for the overall training set simultaneously considering the Q and MCC values. An improvement in the RF model prediction accuracies (Q = 0.821–1 and MCC = 0.64–1) was achieved for these three clusters of the training set, when compared with the prediction accuracy obtained for all the molecules of the training set (Q = 0.811 and MCC = 0.625). For the clusters II and V-VII, lower prediction accuracies were obtained, Q = 0.6–0.842 and MCC = 0.234–0.574. Interestingly, the best achieved predictions for structural clusters I and II were related to the best performance obtained for the active class prediction, with SE values of 1 and 0.889, respectively, compared to the SE value of 0.842 for all training sets. For example, for the test set, the average of the Prob_active (a_Prob_active) obtained by the active molecules predicted by the model as active, i.e., true positive (TP), was 0.59, which compares with the value of a_Prob_active of 0.54 obtained by the predicted molecules by the model as false positives (FP). The same relationship was obtained for molecules predicted as true negatives (FN) and false negatives (FN), with an a_Pro_active of 0.44 and 0.48, respectively. Additionally, it appears that, with a Prob_active higher than 0.59, there was no error in the prediction and all molecules predicted as active were active.

### 2.3. Analysis of Fingerprints and Descriptors Identified as Relevant for Modeling the Antifouling Activity

**Figure 1:**
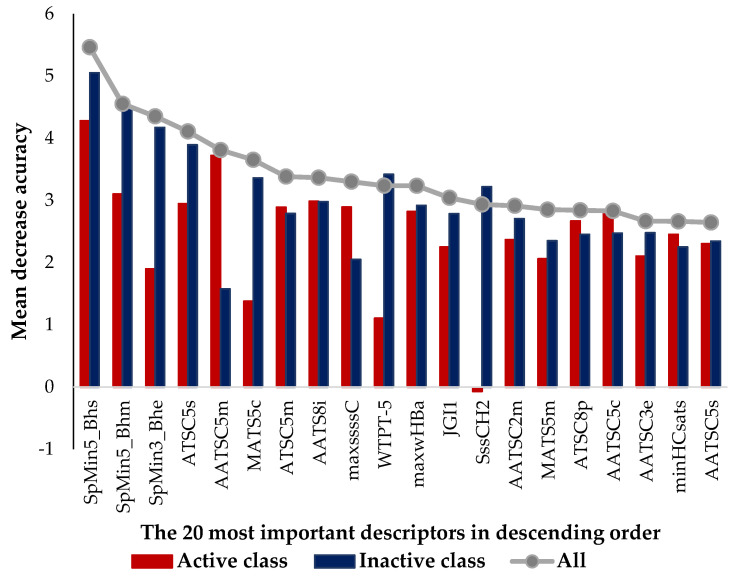
The twenty most important 1D&2D +RDF descriptors selected in RF classification models, where the first three descriptors in terms of importance are three Burden-modified eigenvalue descriptors weighted by relative I-state, mass and Sanderson electronegativities, respectively; there are several Broto–Moreau autocorrelations 4th–5th, 7th–8th, 14th, 16th–18th, 20th weighted by I-state, mass, mass, first ionization potential, mass, polarizabilities, charge, Sanderson electronegativities and I-state; two Moran autocorrelation descriptors, 6th and 15th weighted by charge and mass, respectively; four electrotopological state atom type descriptors, 9th (>C<), 11th (weak hydrogen bond acceptors), 13th (-CH2-), 19th (H bonded to B, Si, P, Ge, As, Se, Sn or P); one PaDEL weighted path descriptor, 10th (sum of path lengths starting from nitrogens); and one topological charge descriptor, 12th (mean topological charge index of order 1).

The selected 200 descriptors included 164 1D&2D (115 topological descriptors, 48 count type descriptors and one constitutional descriptor (Mannhold LogP, logarithm of the octanol–water partition coefficient)) and 36 RDF 3D descriptors (12 of type a (a positive and a negative charge), 12 of type b (two positive charges) and 12 of type c (two negative charges)). The 1D&2D descriptors comprised 72 autocorrelation topological descriptors, which were 50 Broto–Moreau, 12 Moran and 10 Geary autocorrelation descriptors, weighted by mass, charges, van der Waals volumes, Sanderson electronegativities, polarizabilities, first ionization potential or I-state. Other topological descriptors, such as 6 Barysz matrices, 24 Burden-modified eigenvalues, 1 Detour matrix, 2 MDEs, 2 path counts, 3 topological charges, 3 distance matrices, 1 walk count descriptor and 1 weighted path descriptor, were also presented. The count type descriptors included 28 electrotopological state atom types, 10 extended topochemical atoms and 10 information content descriptors. A comparison of the best twenty 1D&2D + RDF molecular descriptors selected by descriptor importance of RF was used to build the QSAR classification models, which are presented in Table 3 and Table 4, and these were analyzed and are presented in Figure 1.

Interestingly, no 3D RDF descriptor appeared in the list of the twenty most important descriptors and the first RDF descriptor appeared only in the 30th position (two positive charges). Moreover, there were only seven out the twenty most important descriptors that were more relevant in discriminating the active class, namely AATSC5m (5th), ATSC5m (7th), AATS8i (8th), maxssssC (9th), ATSC8p (16th), AATSC5c (17th) and minHCsats (19th). Of the nine Broto–Moreau autocorrelation descriptors existing in the list of the top 20, five of them were more relevant to discriminate the active class and, on the other hand, they also presented a lag higher than or equal to 5, which was related to a greater distance between the structural features of interest. In contrast, the four Broto–Moreau autocorrelation descriptors that were more relevant for the inactive class presented a lag lower than or equal to 5. The three most important descriptors in the top 20 list were three Burden-modified eigenvalue descriptors and all of them were most relevant in the inactive class discrimination. This eigenvalue was suggested as an index of molecular branching, the smallest values corresponding to chain graphs (SpMin3_Bhe) and the highest to the most branched graphs (SpMin5_Bhs and SpMin5_Bhm) [53]. A very interesting behavior was observed with the two electrotopological state atom types, maxssssC (maximum atom-type E-state: >C<) and SssCH2 (sum of atom-type E-state: -CH_2_-), which were more relevant for the active and inactive classes, respectively. The maxssssC descriptor encodes the maximum number of quaternary or asymmetric carbon atoms and could be seen as encoding structural complexity. On the other hand, the SssCH2 descriptor encoded the saturation of the molecule. Another very important descriptor to discriminate mainly the inactive class is the PaDEL weighted path descriptor, WTPT-5, which is the sum of all path weights starting from nitrogen atoms, revealing nitrogen-specific branching information. In agreement with the present work, the two QSAR studies reported by Almeida et al. highlighted the descriptors related to the branching, complexity and the influence of the molecule’s interatomic distance for the modeling of the antifouling activity [45,46].

### 2.4. Application of the In Silico Antifouling QSAR Model in Virtual Screening

A virtual screening campaign was carried out to search for new lead-like antifouling inhibitors. The best QSAR model, the RF model, was selected for the virtual screening procedure using 14,492 MNPs from Prof. Encinar’s website and 14 MNPs in the pharmaceutical pipeline (eight approved drugs and six MNPs in Phase II and III of clinical trials). The antifouling virtual screening of the MNP library in the pharmaceutical pipeline allowed us to assess the possibility of repurposing drugs of marine origin. Of these 14 MNPs from the pharmaceutical pipeline, only one MNP in Phase II of clinical trials presented activity against AChE, GTS-21 (DMXBA), a derivative of the NP, 2,4-dimethoxybenzylidene anabaseine dihydrochloride. There were 13,902 MNPs that were predicted to be active by the best QSAR model, of which 8349 MNPs were predicted to be active with a Prob_active greater than 0.59 (limit defined for the test set for which there are no prediction errors). From these MNPs, 5 (one approved drug and four MNPs in Phase II and III of clinical trials) and 8344 MNPs were from the pharmaceutical pipeline and from Encinar’s database, respectively. Interestingly, of the five MNPs from the MNP pharmaceutical pipeline predicted to be active with the highest Prob_active was DMXBA with a value of 0.658. A more demanding limit has been defined for the CADD structure-based approach: all the MNPs from the virtual screening libraries that were predicted as belonging to the active class with a Prob_active greater than or equal to 0.68 were selected for molecular docking experiments. In the CADD structure-based method, the 125 MNPs selected by the QSAR classification approach were screened by molecular docking against acetylcholinesterase enzyme (AChE). 

**Figure 2:**
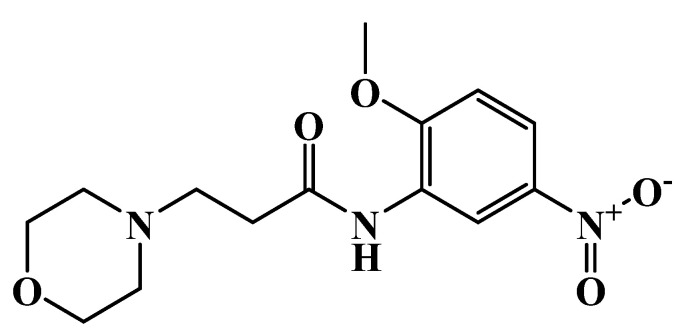
Chemical structure of the morpholine derivative.

The list of eleven lead-like AChE inhibitors against antifouling activity generated from the AChE homology virtual screening, which were experimentally screened in in vitro and micro- and macrofouling assays reported by Arabshahi et al. [50], was used in this study as a second virtual screening library (Supplementary Data, Table S5). Only one out of the eleven lead-like AChE inhibitors was predicted to have antifouling activity with a Prob_active higher than 0.59 (Table S5), the morpholine derivative (Figure 2), in which experimental antifouling activity IC_50_ = 16 μg/mL was reported (51.7 μM) [50]. However, none of the eleven compounds passed the established threshold, which was more demanding (Prob_active ≥ 0.68), to be selected for the molecular docking experiments.

### 2.5. Molecular Docking against AChE Enzyme

The 125 MNPs from Encinar’s database selected by the QSAR classification approach were screened by molecular docking against AChE enzyme (PDB ID: 6TT0) [54]. The antifouling agents, synoxazolidinone A, synoxazolidinone C and donepezil, known as AChE inhibitors [28], were used as positive controls and the phenolic derivative that was predicted to not have antifouling activity in virtual screening was used as a negative control in the molecular docking experiments. A list of virtual screening hits comprising 19 MNPs was approved based on molecular docking experiments, in which a threshold of ΔGB ≤ −7 kcal/mol was established for predicting the affinity between AChE and selected MNPs. To prioritize the best marine drug-like leads as antifouling AChE inhibitors from the list of 19 selected MNPs by the antifouling QSAR model and molecular docking of AChE enzyme, the absorption, distribution, metabolism, excretion and toxicity (ADMET) properties were predicted via in silico methods using the pKCSM software (http://biosig.unimelb.edu.au/pkcsm/, accessed on 25 October 2021) [55]. Sixteen MNPs, a macrocyclic lactam (CAS 156310-18-8), seven macrocyclic alkaloids (CAS 126622-63-7, 126622-64-8, 156310-18-8, 155944-26-6, 157536-35-1, 105305-54-2 and 105418-77-7), seven indole derivatives (CAS 142677-10-9, 134029-43-9, 134029-44-0, 134029-45-1, 142677-09-6, 223596-72-3, 134779-34-3) and a pyridine derivative (CAS 59697-14-2) were proposed as marine drug-like leads as antifouling AChE inhibitors. Three MNPs were excluded due to their predicted toxicity to fish, namely against flathead minnows. The Autodock Vina software (http://vina.scripps.edu/, accessed on 25 October 2021) [56] was used to perform the flexible virtual screening of the 125 MNPs to find the most favorable binding interactions, and the calculated free binding energies by the set of search space coordinates are reported in Table 6 for the 16 MNPs selected, and the positive (synoxazolidinone A and C; donepezil, an AChE inhibitor used for Alzheimer disease therapy) and the negative (phenolic derivative derivative) controls.

**Figure 3:**
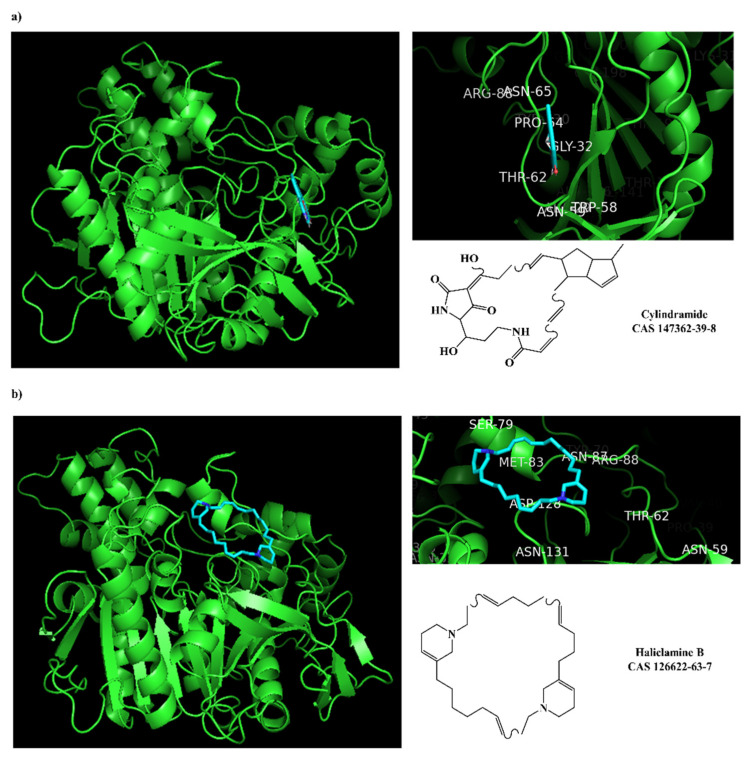
Interaction profiles of the best-docked poses for the two hits (**a**) cylindramide and (**b**) haliclamine B.

The prediction of the ADMET properties of the sixteen selected MNPs by the antifouling QSAR model and molecular docking of AChE enzyme is presented in Table S1, in the Supplementary Materials. In Figure 3, the interaction profile of the best-docked pose for the two most probable lead-like antifouling AChE inhibitors, a lactam derivative—cylindramide—and a macrocyclic alkaloid—haliclamine B—is represented.

**Figure 4:**
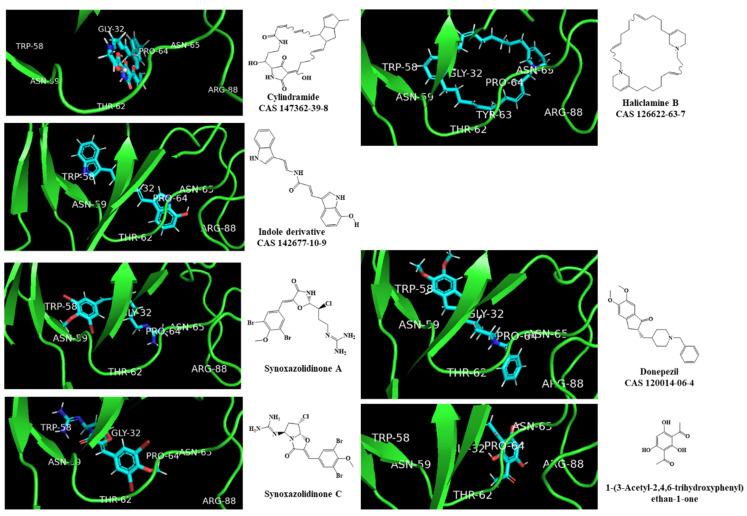
Interaction profiles of the best-docked poses for the two macrocyclic hits (cylindramide and haliclamine B), the best non-macrocycle hit (indole derivative) and the positive (synoxazolidinone A and C; donepezil) and negative (phenolic derivative) controls.

New scoring functions based on more precise physics-based descriptors to better represent the protein–ligand recognition process have been developed. DockThor, a web service for molecular docking simulation (https://dockthor.lncc.br/v2/, accessed on 6 January 2022), was used to perform molecular docking of the two best macrocycle hits (cylindramine and haliclamine B), the best non-macrocycle hit (indole derivative, CAS 142677-10-9) and the positive and negative controls against the AChE enzyme (PDB ID: 6TT0). In DockThor, a set of new empirical scoring functions to estimate protein–ligand binding affinity were developed by explicitly accounting for physics-based interaction terms based on the MMFF94S force field combined with ML [57]. The DockThor scores obtained for the two best macrocycle hits (cylindramine and haliclamine B), the best non-macrocycle hit (indole derivative, CAS 142677-10-9) and the positive (synoxazolidinone A and C; donepezil) and negative (phenolic derivative) controls were −8.508 kcal/mol (−11.3 kcal/mol using Autodock Vina), −7.008 kcal/mol (−8.2 kcal/mol using Autodock Vina), −8.634 kcal/mol (−7.5 kcal/mol using Autodock Vina), −7.749 kcal/mol (−6.5 kcal/mol using Autodock Vina), −7.56 kcal/mol (−6.7 kcal/mol using Autodock Vina) and −6.416 kcal/mol (−5.1 kcal/mol using Autodock Vina), respectively. The interaction profiles of the best-docked poses predicted by DockThor for the two best macrocycle hits, the best non-macrocycle hit and the positive and negative controls are presented in Figure 4.

The peripheral anionic site (PAS) of AChE is composed of five residues (TYR-70, ASP-72, TYR-121, TRP-279 and TYR-334) and is involved in the allosteric modulation of catalysis at the active center [46]. This site is the target of various anti-cholinesterase inhibitors. In this work, other residues (e.g., ARG-88, ASN-65, PRO-64, GLY-32, THR-62, TRP-58 and ASN-59) forming the hydrophobic interactions in the PAS pocket are highlighted in Figure 3 and Figure 4. The binding of donepezil to the PAS of AChE is in accordance with its proposed peculiar inhibitory mechanism, which involves a reversible double-binding site interaction with the catalytic anionic site and PAS of the enzyme [54]. Unlike our approach and in other reported studies [46,54], Arabshahi et al. [50] performed a virtual screening by molecular docking of AChE at the catalytic anionic site and not at the PAS. Although none of the 11 reported compounds [50] passed the QSAR model threshold to be subjected to molecular docking, we still performed the molecular docking and the docking scores are presented in Table S5 (Supplementary Data). It was verified that none of these compounds exceeded the established threshold in the molecular docking experiments, ΔGB ≤ −7 kcal/mol.

## 3. Materials and Methods

### 3.1. Data Sets/Selection of Training and Test Sets

The antifouling data set comprising 142 molecules, 63 and 79 organic molecules, was extracted from the ChEMBL (https://www.ebi.ac.uk/chembl/, accessed on 21 July 2021) [58] and by searching in the literature indexed in the Web of Science Core Collection until June 2021, respectively. The ChEMBL data set was obtained by searching for marine organisms with antifouling activity, such as barnacles (e.g., *Balanus amphitrite*), mussels (e.g., *Mytilus galloprovincialis*), bushy bryozoan (e.g., *Bugula neritina*) and marine algae (e.g., *Ulva conglobata*). The antifouling activity was classified using two activity classes: (A, active)—inhibition % > 52% and EC_50_, IC_50_ ≤ 25 μg/mL; (B, inactive)—inhibition % ≤ 52% and EC_50_, IC_50_ > 25 μg/mL. After collecting these data sets, the duplicates were removed based on the IUPAC international chemical identifier (InChI) codes; however, the chirality was considered, and racemic compounds (or cases where no stereochemistry was indicated) were considered as one of the possible stereoisomers. Thereafter, the final data set comprised 141 organic molecules and was divided into a training set comprising 127 molecules (class A, 57 molecules and class B, 70 molecules) and a test set comprising 14 molecules (class A, 6 molecules and class B, 8 molecules). The partitioning of the data set into training and testing sets was performed randomly according to the composition of the antifouling classes (active and inactive). The composition of the 10 structural categories shown in Table 1 was not considered. The built QSAR models were developed and externally validated using the training and test sets, respectively.

The virtual data set comprised 14,492 MNPs from Prof. Encinar’s website (http://docking.umh.es/downloaddb, accessed on 25 October 2021) saved in the MDL SDF data format and 14 MNPs from the pharmaceutical pipeline set (eight approved drugs and six MNPs in Phase II and III of clinical trials). Three duplicates with the training and test sets were removed and the final virtual data set comprised 14,503 molecules.

A second virtual library comprising eleven lead-like AChE inhibitors against antifouling activity reported by Arabshahi et al. [50] was also used.

SMILES strings of the data sets, and the corresponding experimental and predicted activities, are available as Supplementary Data, Tables S2, S3 and S5.

### 3.2. Calculation of Descriptors

The molecular structures of molecules in all data sets were standardized by normalizing tautomeric and mesomeric groups and by removing small disconnected fragments using the JChem Standardizer tool, version 5.7.13.0 (ChemAxon Ltd., Budapest, Hungary). The optimization of the three-dimensional molecular structures was carried out with CORINA version 2.4 (Molecular Networks GmbH, Erlangen, Germany). PaDEL-Descriptor (Pharmaceutical Data Exploration Laboratory, Singapore) version 2.21 (http://www.yapcwsoft.com/dd/padeldescriptor/, accessed on 21 July 2021) [52] was used to calculate empirical molecular fingerprints (FPs) and 1D&2D molecular descriptors. FPs of various types were calculated and exploited to build QSAR models, namely 166 MACCS (MACCS keys), 307 Substructure (presence and count of SMARTS patterns for Laggner functional group classification—Sub), 881 PubChem fingerprints (ftp://ftp.ncbi.nlm.nih.gov/pubchem/specifications/pubchem_fingerprints.txt, accessed on 21 July 2021), 1024 CDK (circular fingerprints) and 1024 CDK extended (Ext circular fingerprints with additional bits describing ring features). The 1D&2D molecular descriptors comprised descriptors of various types, including electronic, topological and constitutional descriptors, in a total of 1376 descriptors. Radial distribution function (RDF) pair descriptors [59] and 3D RDF descriptors were calculated by sampling the function of Equation (1) at 128 equally distributed values of *r* between 0 and 12.8 Å:

(1) $$RDF\left(r\right)=\overset{N-1}{\underset{i=1}{\sum }}\overset{N}{\underset{j=1+1}{\sum }}p_{i}p_{j}e^{-B\;{\left(r-r_{ij}\right)}^{2}}$$

where N is the number of atoms in the molecule, p_i_ is the charge of atom i, B is a fuzziness parameter (it was 100 in this study), and r_ij_ is the 3D distance between atoms i and j. The *RDF* descriptors were separated into three sets of 128 descriptors per pair of atoms with (a) one positive and one negative charge, (b) two positive charges and (c) two negative charges. The partial atomic charges—natural bond orbital (NBO) partial atomic charges—were estimated using an ML tool developed by Aires-de-Sousa and co-workers (http://joao.airesdesousa.com/charges, accessed on 21 July 2021) [60].

### 3.3. Selection of Descriptors and Optimization of QSAR Models

In the quest for QSAR models with the minimum possible number of descriptors, descriptor selection was performed based on the importance of descriptors assessed by the RF (computeAttributeImportance) algorithm [51] implemented in the R program [61]. Selection of descriptors was accomplished using this procedure, with the importance of descriptors assessed by RF within an OOB methodology using the 12, 25, 50, 100, 150, 200 and 250 most important descriptors and RF algorithm as an ML technique employing the following statistical metrics: true positives (TP), true negatives (TN), false positives (FP), false negatives (FN), sensitivity (SE, prediction accuracy for active antifouling molecules), specificity (SP, prediction accuracy for inactive antifouling molecules), overall predictive accuracy (Q) and Matthews correlation coefficient (MCC).

### 3.4. Class Balancer

In general, class imbalance is more demanding for ML algorithms and this imbalance introduces a bias due to their preference for the majority class [62]. Our antifouling activity training set was unbalanced, and the imbalance ratio was 1:1.22 for the A: active and B: inactive classes, respectively. To solve this problem, the classes were balanced using the RF *sampsize* parameter with R version 3.6.1. [61]. This parameter was set to be of the same size as the minority class (active class). With this parameter, some molecules belonging to the minority class were used more than once.

### 3.5. Machine Learning (ML) Methods

#### 3.5.1. Random Forest (RF)

The RF model [51,63] was built from a set of unpruned classification trees, which were created using bootstrap samples from the training set. For each individual tree, the best split at each node was defined using a randomly selected subset of descriptors. Each of the individual classification trees was created using different training and validation sets. The final prediction of the model resulted from the majority vote of classification trees in the forest. Model performance was evaluated internally with the prediction error for molecules left out in the bootstrap procedure (OOB estimation). The method quantifies the importance of a descriptor by the increase in misclassification that occurs when descriptor values are randomly permuted, correlated with the mean decrease in the precision parameter. RFs also assigned a probability to every prediction based on the number of votes obtained by the predicted class. RFs were grown with the R program [61], version 3.6.1, using the random forest library [64]. As a result of the nature of the two-class imbalance, this problem was alleviated by defining the class weight ranges of 1–57 and 1–57 for classes A and B, respectively, using the *sampsize* parameter.

#### 3.5.2. Support Vector Machines (SVMs)

SVMs [65] map the training data into a hyperspace through a nonlinear mapping (a boundary or hyperplane) and then separate the classes of objects in this space. The examples of the training set—the support vectors—allowed us to position the boundary. To transform data into a hyperspace where classes become linearly separable, kernel functions were used. In this study, SVMs were implemented with Scikit-learn [66] using the LIBSVM package [67]. The type of SVM was set to C-SVM-classification and the radial basis function was used for the kernel function. Hyperparameter tuning was performed using ten-fold cross-validation with the GridSearchCV tool. C and γ values varied in the range of 1 × 10^−2^ to 1 × 10^13^ and 1 × 10^−9^ to 1 × 10^13^, respectively. In total, 10,000 experiments were performed. The C and γ values were finally set to 1 × 10^7^ and 1 × 10^−8^, respectively, and the other parameters were used with default values. To alleviate the imbalanced two-class problem, the class_weight parameter was set to be “balanced”, in which the smaller class was replicated until it had as many molecules as in the larger one class.

#### 3.5.3. Deep Learning Multilayer Perceptron Networks (_d_MLP)

The feed-forward neural networks were implement using the open-source software library Keras [68] version 2.2.5 based on the Tensorflow numerical backend engine [69]. These popular software tools, written in Python, make it easy to develop and apply deep neural networks; however, the main challenge in applying _d_MLP is the design of an adequate network architecture. After several experiments, the final optimal hyperparameter settings were selected for our study based on 10-fold cross-validation experiments with the training set and are listed in Table 7.

### 3.6. Molecular Docking

The virtual screening using the best QSAR model, the RF classification model using the 200 most important 1D&2D + RDF molecular descriptors, allowed the prioritization of a list of the 125 MNP virtual screening hits. OpenBabel software (version 2.3.1, freely available under an open-source license from http://openbabel.org, accessed on 21 July 2021) [70] was used to convert mol2 files into PDBQT files. PDBQT files were used for coupling to the AChE enzyme with Autodock Vina (version 1.1, Center for Computational Structural Biology, Scripps Research Institute, CA, USA) [56]. The macromolecule coupling target was the AChE enzyme from *Tetronarce californica* (PDB ID: 6TT0) [54]. Water molecules, carbohydrate molecules and ligands (1R, 3S-cis- and 1S, 3R-cis-donepezil derived enantiomers) were removed from 6TT0 [54] prior to docking using AutoDockTools (http://mgltools.scripps.edu/, accessed on 21 July 2021). During enzyme preparation, GTT0, explicit hydrogen atoms and Gasteiger charges for each atom were added. Autodock Vina performed a flexible molecular docking in which the target’s conformation was considered a rigid unit while the ligands were flexible and adaptable to the target. Autodock Vina looked for the lowest binding affinity conformations and returned ten different conformations for each ligand. The search space coordinates of the AChE enzyme were maximized to allow the entire macromolecule to be considered for docking. The search space coordinates were center X: 25.179 Y: 72.212 Z: 281.175; dimensions X: 20,000 Y: 20,000 Z: 20,000. AchE enzyme ligand tethering was performed by regulating the parameters of the genetic algorithm (GA), using 10 runs of the GA criteria. DockThor, a web service for molecular docking simulation (https://dockthor.lncc.br/v2/, accessed on 6 January 2022), was used to perform molecular docking of the two best macrocycle hits (cylindramine and haliclamine B), the best non-macrocycle hit (indole derivative, CAS 142677-10-9) and the positive and negative controls against AChE enzyme (PDB ID: 6TT0) [57]. The search space coordinates were center X: 25.179 Y: 72.212 Z: 281.175; dimensions X: 20,000 Y: 20,000 Z: 20,000. AChE enzyme ligand tethering was performed by regulating the parameters of the GA, using 12,750 and 500,000 runs, population size and number of evaluations of the GA criteria, respectively.

The docking binding poses were visualized with PyMOL Molecular Graphics System, Version 2.0 (Schrödinger, LLC). Docking scores of 125 virtual hits against the AChE enzyme are shown in Table S4, Supplementary Data.

## 4. Conclusions

A CADD approach relying on ligand- and structure-based methodologies was successfully used to predict new inhibitory MNPs against antifouling AChE. Two MNPs, cylindramide (CAS 147362-39-8) and haliclamine B (CAS 126622-63-7), were proposed as the most promising marine drug-like leads as antifouling AChE inhibitors. To the best of our knowledge, the CADD ligand-based study using a QSAR classification model, developed here in this study, is the largest study ever performed with regard both to the number of molecules involved and to the number of structural families involved in the modeling of the antifouling activity, and the best model achieved an overall predictive accuracy score of up to 71% for both test and training sets. In future works, the proposed sixteen marine drug-like leads against antifouling AChE enzyme may be validated experimentally. These results enabled us to build virtual libraries of marine-derived drug-like leads, which may be virtually screened using the best antifouling QSAR model and molecular docking against the AChE enzyme. In addition, for MNPs that are experimentally confirmed to have antifouling activity, the AChE inhibitory mechanism will be studied to determine the type of action, e.g., reversible interaction with both the catalytic anionic site and the PAS, sterically blocking ligands from entering and leaving the active site gorge and allosteric alteration of the catalytic triad conformation.

## Figures and Tables

**Table 1:** Structural clusters and antifouling activity class counts within the seven structural clusters.

Clusters ^1^	# ^2^ (Active Class)		Average MW (Da) ^3^		Average ALogP ^4^	
	Tr	Te	Tr	Te	Tr	Te
I—acyclic derivative 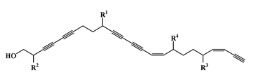	11 (11)	0 (0)	361.65	0	2.86	0
II—*O*-heterocyclic derivative 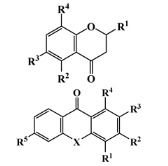	28 (9)	3 (1)	328.09	334.64	3.18	3.22
III—*N*-heterocyclic derivative 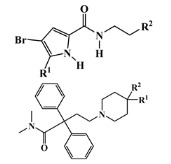	19 (14)	1 (0)	363.92	493.04	2.50	3.65
IV—terpenoid derivative 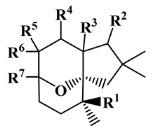	22 (5)	6 (3)	264.64	341.76	3.00	4.49
V—diketopiperazine derivative 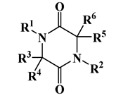	15 (10)	3 (2)	392.54	415.15	3.06	3.10
VI—chalcone derivative 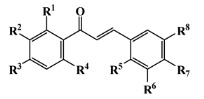	16 (3)	0 (0)	352.37	0	4.56	0
VII—miscellaneous	16 (5)	1 (0)	1164.53	975.69	−0.88	−1.57

^1^ Cluster code and chemical structure of the cluster scaffold. ^2^ Number of molecules in the training (Tr) and the test (Te) sets. ^3^ Molecular weight (MW) within the cluster for the training and test sets. ^4^ Octanol–water partition coefficient prediction within the cluster for the training and test sets.

**Table 2:** Evaluation of the predictive performance of FPs and 1D&2D molecular descriptors for modeling the antifouling activity using the RF algorithm for the training set with an OOB estimation. The best models are highlighted in bold.

Descriptors (#)	TP ^1^	TN ^2^	FN ^3^	FP ^4^	SE ^5^	SP ^6^	Q ^7^	MCC ^8^
MACCS (166) ^9^	41	51	16	19	0.719	0.729	0.724	0.446
Sub (307) ^9^	41	53	16	17	0.719	0.757	**0.740**	**0.476**
PubChem (881) ^9^	43	48	14	22	0.754	0.686	0.717	0.438
CDK (1024) ^9^	42	47	15	23	0.737	0.671	0.701	0.406
ExtCDK (1024) ^9^	41	49	16	21	0.719	0.700	**0.709**	**0.417**
1D&2D (1376)	40	53	17	17	0.702	0.757	**0.732**	**0.459**

^1^ True positive. ^2^ True negative. ^3^ False negative. ^4^ False positive. ^5^ Sensitivity, the ratio of true positive to the sum of true positive and false positive. ^6^ Specificity, the ratio of true negative to the sum of true negative and false negative. ^7^ Overall predictive accuracy, the ratio of the sum of true positive and true negative to the sum of true positive, true negative, false positive and false negative. ^8^ Matthews correlation coefficient. ^9^ Fingerprints, FPs.

**Table 3:** Evaluation of the predictive performance of RDF descriptors and descriptor selection for modeling the antifouling activity using the RF algorithm for the training set with an OOB estimation. The best models are highlighted in bold.

Model	#	SE ^1^	SP ^2^	Q ^3^	MCC ^4^
Sub + RDF	691	0.667	0.714	0.693	0.380
Selection ^5^	50	0.667	0.714	0.693	0.380
Selection ^5^	100	0.684	0.757	0.724	0.442
Selection ^5^	150	0.702	0.786	**0.748**	**0.489**
Selection ^5^	200	0.684	0.757	0.724	0.442
ExtCDK + RDF	1408	0.667	0.743	0.709	0.410
Selection ^5^	12	0.754	0.729	0.740	0.481
Selection ^5^	25	0.737	0.786	**0.764**	**0.523**
Selection ^5^	50	0.702	0.771	0.740	0.474
Selection ^5^	100	0.684	0.771	0.732	0.457
1D&2D + RDF	1760	0.719	0.714	0.717	0.432
Selection ^5^	50	0.807	0.800	0.803	0.605
Selection ^5^	100	0.825	0.786	0.803	0.607
Selection ^5^	150	0.807	0.800	0.803	0.605
Selection ^5^	200	0.842	0.786	**0.811**	**0.625**
Selection ^5^	250	0.772	0.800	0.787	0.571

^1^ Sensitivity, the ratio of true positive to the sum of true positive and false positive. ^2^ Specificity, the ratio of true negative to the sum of true negative and false negative. ^3^ Overall predictive accuracy, the ratio of the sum of true positive and true negative to the sum of true positive, true negative, false positive and false negative. ^4^ Matthews correlation coefficient. ^5^ The descriptor selection was evaluated based on the importance assigned by the RF model with the R program.

**Table 4:** Exploration of different ML algorithms using the 200 selected descriptors.

Model	SE ^1^	SP ^2^	Q ^3^	MCC ^4^
RF	0.667	0.750	0.714	0.417
SVM	0.830	0.500	0.643	0.344
_d_MLP	0.670	0.750	0.714	0.417

^1^ Sensitivity, the ratio of true positive to the sum of true positive and false positive. ^2^ Specificity, the ratio of true negative to the sum of true negative and false negative. ^3^ Overall predictive accuracy, the ratio of the sum of true positive and true negative to the sum of true positive, true negative, false positive and false negative. ^4^ Matthews correlation coefficient.

**Table 5:** The predictions of the best RF model by the seven structural clusters for the training and test sets. The best models are highlighted in bold.

Cluster	#	SE ^1^	SP ^2^	Q ^3^	MCC ^4^
Training set					
I	11	1.000	-	**1.000**	**1.000**
II	28	0.889	0.789	**0.821**	**0.640**
III	19	1.000	0.400	0.842	0.574
IV	22	0.800	0.941	**0.909**	**0.741**
V	15	0.900	0.000	0.600	-
VI	16	0.000	1.000	0.813	-
VII	16	0.400	0.812	0.688	0.234
All		0.842	0.786	0.811	0.625
Test set					
II	3	1.000	1.000	**1.000**	**1.000**
III	1	-	1.000	**1.000**	**1.000**
IV	6	0.333	1.000	0.667	0.447
V	3	1.000	0.000	0.667	-
VII	1	-	0.000	0.000	-
All		0.667	0.750	0.713	0.417

^1^ Sensitivity, the ratio of true positive to the sum of true positive and false positive. ^2^ Specificity, the ratio of true negative to the sum of true negative and false negative. ^3^ Overall predictive accuracy, the ratio of the sum of true positive and true negative to the sum of true positive, true negative, false positive and false negative. ^4^ Matthews correlation coefficient.

**Table 6:** Structures and calculated free binding energies (∆G_B_, in kcal/mol) of the sixteen selected MNPs, the positive (synoxazolidinone A and C; donepezil) and negative (phenolic derivative) controls.

CAS	Chemical Structure	Name/Structural Category	Natural Source	Prob_A	∆G_B_ (kcal/mol) ^1^
147362-39-8	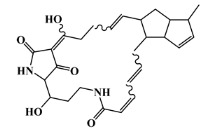	cylindramide/lactam	marine sponge ^2^	0.684	−11.3
126622-63-7	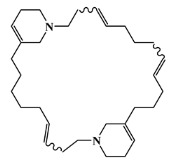	haliclamine B/macrocyclic alkaloid	marine sponge ^3^	0.682	−8.2
126622-64-8	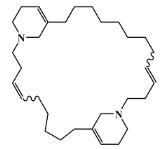	haliclamine A/macrocyclic alkaloid	marine sponge ^3^	0.682	−7.8
156310-18-8	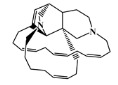	ingamine B/macrocyclic alkaloid	marine sponge ^4^	0.682	−7.8
155944-26-6	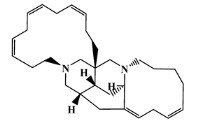	madangamines A/macrocyclic alkaloid	marine sponge ^4^	0.694	−7.7
105305-54-2	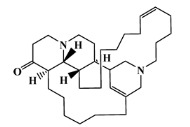	serain 3/ macrocyclic alkaloid	marine sponge ^5^	0.686	−7.5
142677-10-9	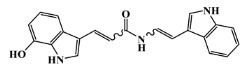	chondriamide B/indole	red alga ^6^	0.682	−7.5
134029-43-9	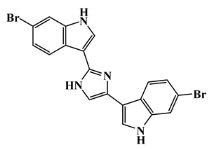	nortopsentin A/indole	marine sponge ^7^	0.702	−7.3
134029-44-0	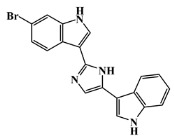	nortopsentin B/indole	marine sponge ^7^	0.698	−7.3
134029-45-1	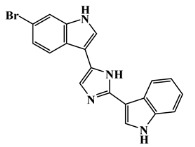	nortopsentin C/indole	marine sponge ^7^	0.700	−7.3
105418-77-7	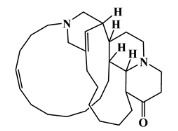	serain 1/ macrocyclic alkaloid	marine sponge ^5^	0.686	−7.2
142677-09-6	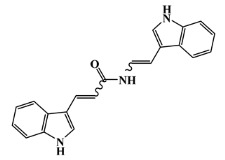	chondriamide A/indole	red alga ^6^	0.682	−7.2
223596-72-3	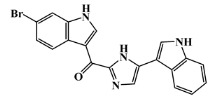	isobromodeoxytopsent/ indole	marine sponge ^8^	0.680	−7.2
134779-34-3	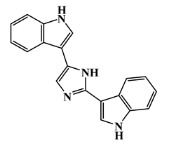	nortopsentin D/indole	marine sponge ^7^	0.688	−7.1
157536-35-1	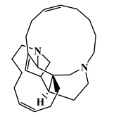	keramaphidin B/macrocyclic alkaloid	marine sponge ^9^	0.684	−7.1
59697-14-2	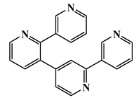	nemertelline/ pyridine	marine worm ^10^	0.680	−7.0
positive control	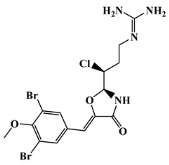	synoxazolidinone A	-	-	−6.5
positive control	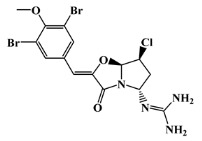	synoxazolidinone C	-	-	−6.7
positive control	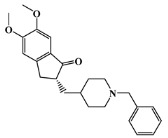	donepezil	-	-	−6.5
negative control	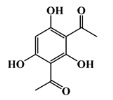	phenolic	-	-	−5.1

^1^ AChE enzyme: center X: 25.435 Y: 69.621 Z: 278.986; ^2^ *Halichondria cylindrata*; ^3^ *Haliclona* sp.; ^4^ *Xestospongia ingens*; ^5^ *Reniera sarai*; ^6^ *Chondria* sp.; ^7^ *Spongosorites ruetzleri* and *Haliclona* sp.; ^8^ *Spongosorites* sp.; ^9^ *Amphimedon* sp.; ^10^ *Amphiporus angulatus*.

**Table 7:** Hyperparameter settings of the best _d_MLP model.

Hyperparameter	Setting
Initializer	Glorot uniform
Number of hidden layers	2
Number of neurons in the 1st and 2nd layers	200
Number of neurons in the 3rd	2
Activation 1st–2nd layers	Relu
Activation 3rd layer	Sigmoid
Batch size	36
Optimizer	Adadelta
Loss	Binary crossentropy
Epochs	100

## Data Availability

Data are contained within the article or Supplementary Material.

## Supplementary Materials

The following data are available online at https://www.mdpi.com/article/10.3390/md20020129/s1, Tables S1–S5 (XLSX). The following files are available free of charge. SMILES strings of the data set (training and test sets), the corresponding experimental and predicted activities are available as Supplementary Materials, Tables S2 and S3, respectively. Moreover, SMILES strings of the 14,492 MNPs from Encinar’s website and MNPs clinical pipeline sets, for the virtual screening data set, the corresponding predicted activities are available as Supplementary Materials, Table S4. Predictions of ADMET properties with in silico methods, using the pKCSM software for a list of 16 selected MNPs by QSAR antifouling model and molecular docking of AChE enzyme are available as Supplementary Materials, Table S1. The list of eleven lead-like AChE inhibitors by Arabshahi et al. [50], the corresponding experimental, predicted activities and docking scores against the AChE enzyme are available as Supplementary Materials, Table S5.
